# Novel Secondary Somatic Mutations in Ewing's Sarcoma and Desmoplastic Small Round Cell Tumors

**DOI:** 10.1371/journal.pone.0093676

**Published:** 2014-08-13

**Authors:** Yunyun Jiang, Vivek Subbiah, Filip Janku, Joseph A. Ludwig, Aung Naing, Robert S. Benjamin, Robert E. Brown, Pete Anderson, Razelle Kurzrock

**Affiliations:** 1 Department of Investigational Cancer Therapeutics (Phase I Clinical Trials Program), Division of Cancer Medicine, The University of Texas MD Anderson Cancer Center, Houston, Texas, United States of America; 2 Department of Pediatrics, The University of Texas MD Anderson Cancer Center, Houston, Texas, United States of America; 3 Department of Sarcoma Medical Oncology, The University of Texas MD Anderson Cancer Center, Houston, Texas, United States of America; 4 From the Department of Pathology, UT Health-Department of Pathology and Laboratory medicine, Houston, Texas, United States of America; 5 Pediatric Hematology/Oncology/BMT, Levine Children's Hospital/Levine Cancer Institute, Charlotte, North Carolina, United States of America; 6 Center for Personalized Cancer Therapy and Division of Hematology & Oncology, University of California San Diego - Moores Cancer Center, La Jolla, California, United States of America; Dana-Farber Cancer Institute, United States of America

## Abstract

**Background:**

Ewing's sarcoma (ES) and desmoplastic small round cell tumors (DSRCT) are small round blue cell tumors driven by an N-terminal containing EWS translocation. Very few somatic mutations have been reported in ES, and none have been identified in DSRCT. The aim of this study is to explore potential actionable mutations in ES and DSRCT.

**Methodology:**

Twenty eight patients with ES or DSRCT had tumor tissue available that could be analyzed by one of the following methods: 1) Next-generation exome sequencing platform; 2) Multiplex PCR/Mass Spectroscopy; 3) Polymerase chain reaction (PCR)-based single- gene mutation screening; 4) Sanger sequencing; 5) Morphoproteomics.

**Principal Findings:**

Novel somatic mutations were identified in four out of 18 patients with advanced ES and two of 10 patients with advanced DSRCT (six out of 28 (21.4%));*KRAS* (n = 1), *PTPRD* (n = 1), *GRB10* (n = 2), *MET* (n = 2) *and PIK3CA* (n = 1). One patient with both PTPRD and GRB10 mutations and one with a GRB10 mutation achieved a complete remission (CR) on an Insulin like growth factor 1 receptor (IGF1R) inhibitor based treatment. One patient, who achieved a partial remission (PR) with IGF1R inhibitor treatment, but later developed resistance, demonstrated a *KRAS* mutation in the post-treatment resistant tumor, but not in the pre-treatment tumor suggesting that the RAF/RAS/MEK pathway was activated with progression.

**Conclusions:**

We have reported several different mutations in advanced ES and DSRCT that have direct implications for molecularly-directed targeted therapy. Our technology agnostic approach provides an initial mutational roadmap used in the path towards individualized combination therapy.

## Introduction

Ewing's sarcoma (ES) and desmoplastic small round cell tumor (DSRCT) are small cell sarcomas characterized by the translocation of chromosome 22 to chromosome 11, resulting in fusion of the *EWSR1* gene to the *FLI1* gene (and several rarer fusion partners) [1], [2] in the case of ES (*EWSR1-FLI1*), and to the *WT1* gene in the case of DSRCT (*EWSR1-WT1*) [3], [4], [5]. While both are mediated by the EWS translocated chimera, the clinical presentation, pathologic features, and response to therapy are different. They have been treated with similar chemotherapy regimens using a multi-disciplinary approach but this does not reflect similar biology. Although genotypically and phenotypically different, historically they have been grouped together based on their shared sensitivity to chemotherapies otherwise used for Ewing's sarcoma. For Ewing's sarcoma, in the United States, the current cytotoxic chemotherapy-based standard of care promoted by the Children's Oncology Group has resulted in steadily improved survival rates over the last four decades for the 60% of ES patients fortunate enough to present with radiographically localized disease [6]. Twenty-three percent of patients present with metastatic disease at diagnosis. Unfortunately, despite equivalent response rates among those who present with metastatic disease, the antineoplastic responses are often short-lived and the 5-year survival of this patient population remains stubbornly in the 30% range [1], [7]. Early phase studies have demonstrated remarkable responses in patients with advanced ES treated with an insulin-like growth factor-1 receptor (IGF1R) antibody, with or without an mTOR inhibitor, albeit in only a small subset (10–14%) of individuals [1], [8], [9], [10], [11], [12], [13]. To date, no predictive biomarkers of IGF-1R response have been identified and only recently have resistance mechanisms, such as shift from the IGF-1R/IGF-1 axis towards IR-A/IGF-2, been identified [11], [14]. For patients with DSRCT, the chemotherapy, radiotherapy, and surgical approaches have not been standardized, given the extreme rarity of the disease. [15], [16], [17]. The most commonly used cytotoxic regimens employ alkylator and anthracycline chemotherapy similar to those administered in Ewing's sarcoma.

Recent identification of molecular mutations such as *BRAF* V600E in some melanomas as well as ALK rearrangements in non-small cell lung cancer has enabled targeted therapies directed toward such anomalies and significantly altered the therapeutic landscape for afflicted patients [18], [19]. While *EWSR1-FLI1* and *EWSR1-WT1* translocations occur in virtually all ES and DSRCT patients, respectively, the resulting fusion proteins have not proved to be druggable targets. Therefore, an alternative approach would be to expand the therapeutic focus to ancillary genetic aberrations infrequently encountered in those tumor types. Toward that end, a recent study using sequence-based genotyping identified mutations in 4% (three of 75 patients or cell lines with ES), including *BRAF*, *CTNNB1*, and *NRAS* [20]. No somatic mutations have been identified in patients (total = 24 samples) with DSRCT [20]. We recently reported germline Protein tyrosine phosphatase delta (PTPRD) mutations in a small pilot study in patients with Ewing's sarcoma [21].

Here we report potential actionable mutations identified in four patients with advanced ES and in two patients with advanced DSRCT. The mutations include *KRAS* (G13N), *PTPRD* (W775D), *GRB10* (Q107stop and V109A), *MET* (T1010I and N375S) and *PIK3CA* (M1040I and G1049S). Correlations with response and resistance to IGF1R inhibitor based regimen in these patients are discussed.

## Patients and Methods

We reviewed 62 consecutive patients with advanced metastatic ES or DSRCT who were referred to the Department of Investigational Cancer Therapeutics (A Phase 1 program) and/or the Department of Pediatrics or Department of Sarcoma at The University of Texas MD Anderson Cancer Center (MD Anderson). Of the 62 patients, 28 patients had tumor archival tissue available that could be analyzed by one of the following methods: 1) next-generation sequencing using the Clinical Laboratory Improvement Amendment (CLIA)-approved FoundationOne platform (Foundation Medicine, Cambridge, MA; 182 genes tested); 2) CLIA-approved mutation screening by Multiplex PCR/Mass Spectroscopy (Knight Cancer Institute, Oregon, Ohio; 53 genes tested); 3) CLIA-approved polymerase chain reaction (PCR)-based single- gene mutation screening in the Department of Pathology at MD Anderson (up to 10 genes tested); 4) next-generation whole exome sequencing in the Center of Targeted Therapy CORE at MD Anderson; 5) Sanger sequencing; 6) immunohistochemical and morphoproteomic analyses of tumor samples collected from patient # 1 were performed, as previously described [11], [22]. Briefly, for patient # 1, morphoproteomics and immunohistochemical probes were used to detect p-ERK1/2 (Thr202/Tyr204) ([Cell Signaling Technology, Beverly, MA] along with negative controls [11], [22]. Testing was done according to tissue availability, test and physicians' choice. This study and all treatments were approved by the MD Anderson Institutional Review Board and conducted in accordance with the MD Anderson Institutional Review Board requirements. The data from this manuscript is based on CLIA certified next generation sequencing from commercially available resources. As such we do not have access to the primary data set. However, data can be made available to researchers upon request. Because this is a retrospective analysis the consent requirements were waived by the MD Anderson Institutional Review Board and informed consents were obtained from patients for collection of samples at the time of screening or enrollment of patients to the clinical trials.

## Results

Among the 28 patients tested for molecular aberrations, 18 had ES and 10 had DSRCT. Four ES patients (22.2%) and two DSRCT patients (20%) had secondary mutations, including *GRB10* (n = 2), *PTPRD* (n = 1), *KRAS* (n = 1), *MET* (n = 2) and *PIK3CA* (n = 1) (Table 1). All patients had metastatic disease. The medium age of the 28 patients at diagnosis was 22 years (range 9 to 52 years). Twenty were men and most had been heavily pretreated with chemotherapy with a median of four prior systemic treatments at the time of tissue acquisition (range, 0 to11). The prior therapies consisted of standard front-line alkylator and anthracycline-based chemotherapy as is standard in the USA and Europe: vincristine, adriamycin, cyclophosphamide, ifosfamide and etoposide. In addition, several patients were treated with additional standard second-line and third -line agents like temozolomide/irinotecan, topotecan/cyclophosphamide and gemcitabine/docetaxel.

**Table 1:** Ewing's sarcoma and Desmoplastic small round cell tumor (DSRCT) patients whose tumors were analyzed for somatic aberrations.

Pt#	Diagnosis	Mutation	Methods used for molecular analysis
**1**	Ewing's	KRAS: G13N	Knight Diagnostics 53 gene panel (Sequenome) Foundation One 182 gene panel (Foundation Medicine), MDACC single gene PCR based assay
**2**	Ewing's	PTPRD:W775stop GRB10:Q107stop	Foundation One 182 gene panel (Foundation Medicine), Next generation sequencing
**3**	Ewing's	GRB10: V109A	Sanger sequencing
**4**	Ewing's	MET: T1010I	MDACC single gene PCR based assay
**5**	Ewing's	none	‘’
**6**	Ewing's	none	‘’
**7**	Ewing's	none	‘’
**8**	Ewing's	none	‘’
**9**	Ewing's	none	‘’
**10**	Ewing's	none	‘’
**11**	Ewing's	none	‘’
**12**	Ewing's	none	‘’
**13**	Ewing's	none	‘’
**14**	Ewing's	none	‘’
**15**	Ewing's	none	‘’
**16**	Ewing's	none	‘’
**17**	Ewing's	none	Foundation One 182 gene panel (Foundation Medicine), Next generation sequencing
**18**	Ewing's	none	MDACC single gene PCR based assay
**19**	DSRCT	MET:N375S	‘’
**20**	DSRCT	PIK3CA:M1040I&G1049S	‘’
**21**	DSRCT	none	‘’
**22**	DSRCT	none	‘’
**23**	DSRCT	none	‘’
**24**	DSRCT	none	‘’
**25**	DSRCT	none	‘’
**26**	DSRCT	none	‘’
**27**	DSRCT	none	‘’
**28**	DSRCT	none	‘’

**Figure 1:**
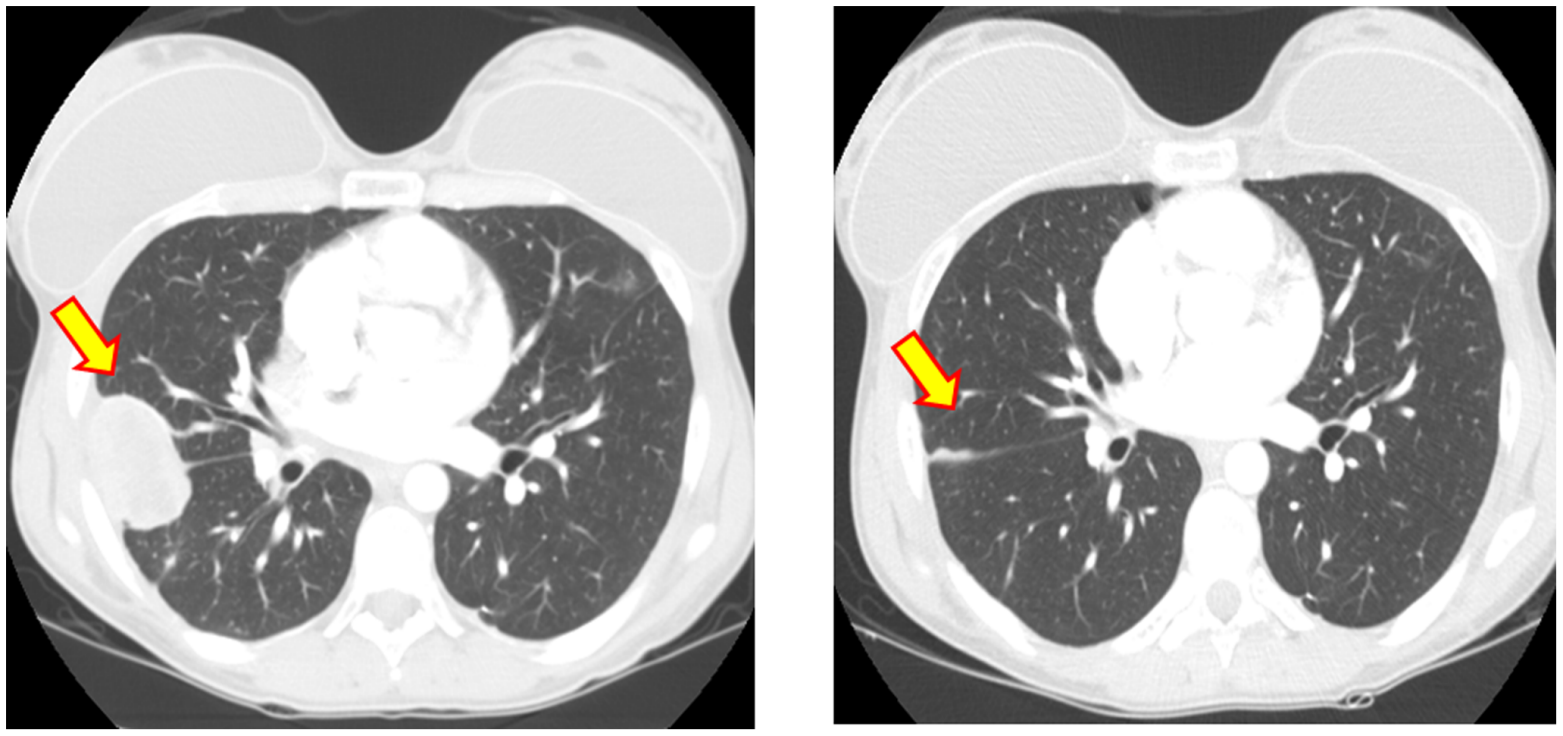
CT of the chest in Case # 2 with Ewing's sarcoma response to single agent IGF1R antibody alone [11]. Left panel shows lung metastases before IGF1R antibody therapy and right panel shows response to therapy after six weeks of treatment [11].

Of interest, both of the patients with *GRB10* mutations (cases #2 and #3, Table 1) responded to therapy with an IGF1R plus mTOR inhibitor and both achieved a complete remission (CR) [10]. One of the patients (case #2) also attained a prior CR with IGF1R inhibitor alone [8], [11], [13] (Figure 1). Case # 2 had been selected for an in-depth molecular analyses with the use of next generation exome sequencing and Foundation One (Foundation Medicine) 182 gene panel interrogation because of her excellent response (CR) after IGF1R inhibitor therapy. Because of the finding of *GRB10* mutation in case #2, mutational analysis of *GRB10* was also performed on tumor from case #3 (who also achieved a CR after IGF1R inhibitor therapy) and, remarkably, was also positive by Sanger sequencing.

**Figure 2:**
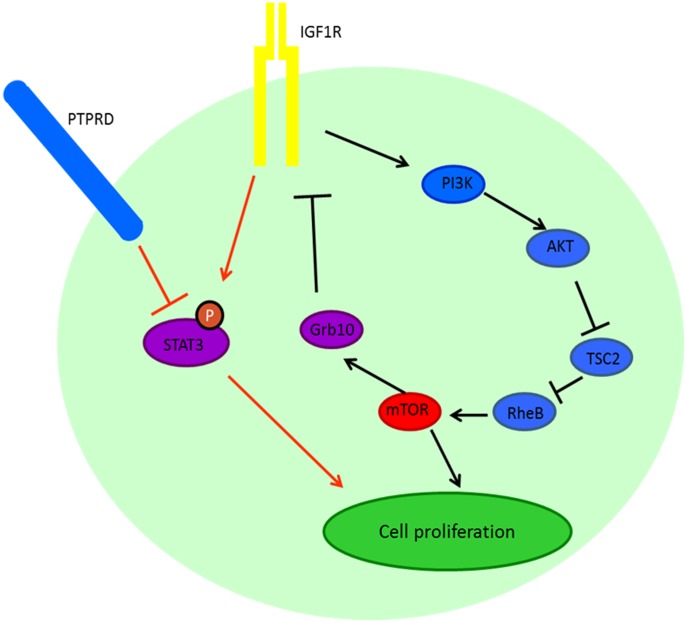
Schematic figure of the IGF1R pathway, regulation of STAT3 phosphorylation and negative feedback of Grb10. IGF1R is one of the regulators for STAT3 phosphorylation while PTPRD dephosphorylates STAT3. STAT3 is recruited to IGF1R for phosphorylation. IGF1R is modulated by several downstream cascades, including PI3K, AKT and mTOR. mTOR phosphorylates Grb10, which in turn inhibits the IGF1R pathway.

The Grb10 protein sends a negative feedback signal to IGF1R, hence inhibiting the IGF1R signal; a loss of function *GRB10* mutation may therefore be expected to be associated with activated IGF1-R signaling (Figure 2) [23], [24]. Case # 2 also demonstrated a protein tyrosine phosphatase receptor type D (PTPRD) mutation that would truncate this protein (Table 1). The PTPRD protein dephosphorylates STAT3, whereas IGF1R is important in STAT3 phosphorylation [21], [25]. A PTPRD mutation would be expected to inhibit STAT3 dephosphorylation, resulting in accumulated phosphorylated STAT3 (Figure 2). Because of the role of IGF1R in enabling STAT3 phosphorylation, inhibiting IGF1R might diminish the up regulation of STAT3 phosphorylation effected by mutated PTPRD. Of interest, increased expression of phospho-STAT3 has been implicated in ES pathogenesis [26].

**Figure 3:**
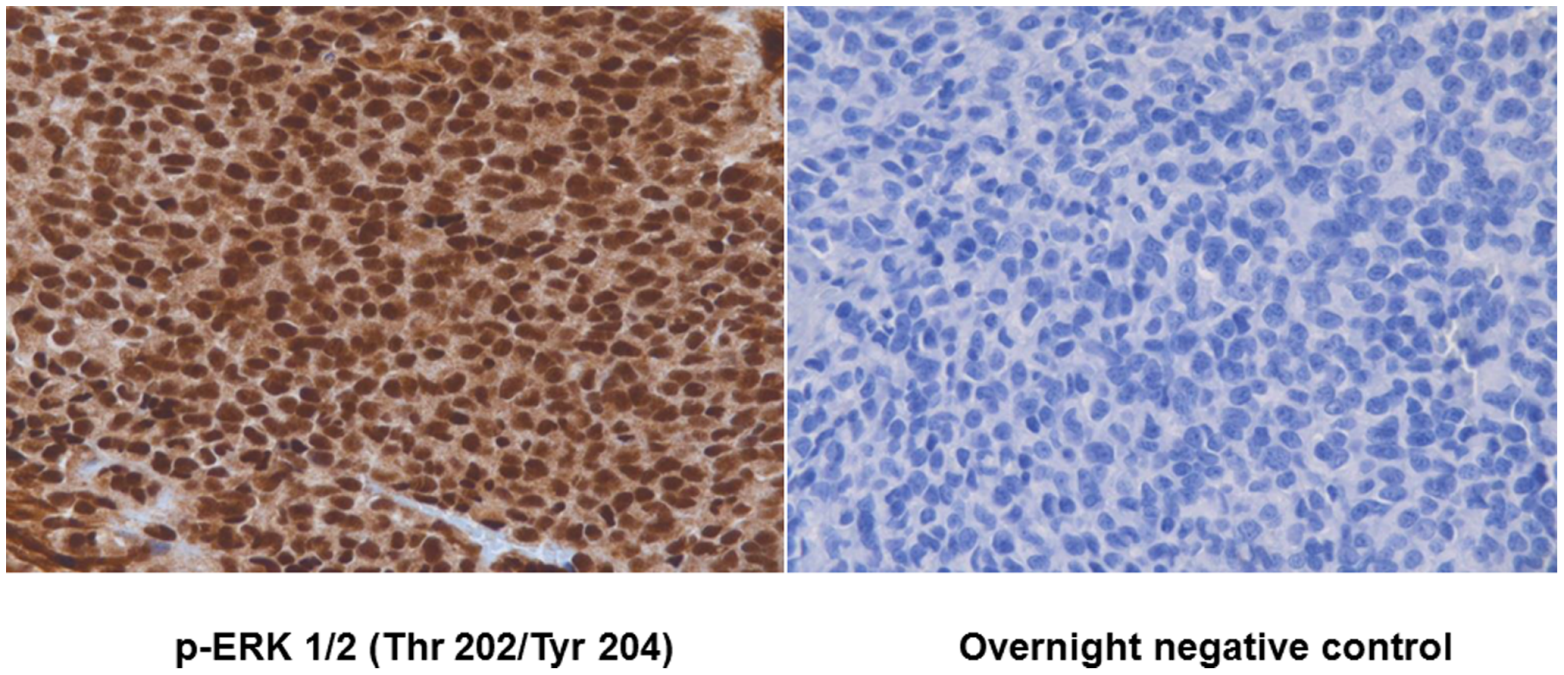
Immunohistochemistry based morphoproteomics of Ewing's sarcoma sample that showed KRAS mutation post IGF1R therapy (Patient 1). The Ras/Raf kinase/extracellular signal-regulated kinase (ERK) pathway was constitutively activated with chromogenic signal observed, up to 3+ in nucleus and ± in the cytoplasm, for p-ERK 1/2 (Thr 202/Tyr 204) (left hand panel) with the overnight negative control (Right hand panel).

One patient (Case # 1, Table #1) received IGF1R/mTOR inhibitor therapy and achieved a partial remission (PR) for 6 months, but developed resistance to the therapy. A *KRAS* mutation was identified in the resistant tumor, but not in the pre-treatment tumor. This was c.37_38GG>AA and was reported in the 53 gene sequenom panel (Knight Diagnostics assay). In addition, immunohistochemistry-based morphoproteomics in the tumor specimen showed that the ras/Raf kinase/extracellular signal-regulated kinase (ERK) pathway was constitutively activated with chromogenic signal observed, up to 3+ in nucleus and ± in the cytoplasm, for p-ERK 1/2 (Thr 202/Tyr 204) (Figure 3). A fourth patient (Table 1, Case # 4), whose tumor demonstrated a *MET* mutation did not receive IGF1R inhibitor therapy. The mutation was detected in codon 1010 (ACT to ATT) of the *MET* gene that would change the encoding amino acid from Thr to Ile (p.T1010I) using a screening assay (PCR-based primer extension analysis).

Of the ten DSRCT patients tested, one patient had a *MET* mutation. Mutation was detected in codon 375 (AAC to AGC) of the *MET* gene that would change the encoding amino acid from Asn to Ser (N375S). The other had two mutations in the *PIK3CA* gene (Table # 1, Case #19 and # 20). Mutation was detected in codon 1040 (ATG to ATA) in exon 20 of the *PIK3CA* gene that would change the encoding amino acid from Met to Ile (M1040I). A second mutation detected in codon 1049 (GGT to AGT) of the *PIK3CA* gene that would change the encoding amino acid from Gly to Ser (G1049S).

## Discussion

Our study shows that 21.4% of patients (6 of 28) with advanced, heavily pretreated Ewing's sarcoma family of tumors (ES, n = 18; DSRCT, n = 10) had secondary somatic molecular aberrations, including mutations in *KRAS*, *PTPRD*, *GRB10*, *MET* and *PIK3CA*. Interestingly, we recently reported germline PTPRD mutations in three out of eight (37.5%) patients with metastatic Ewing's sarcoma [21]. Previous studies by Shukla et al [17] reported molecular aberrations in 3 of 75 patients with ES (4%) and 0 of 24 patients with DSRCT [20]. The higher incidence of somatic mutations in our current study may be due to evolution of technologies. Shukla et al [17] had used a 29 gene panel sequenom assay while we used a range of evolving technologies, including next generation exome sequencing, a 53-gene panel sequenom (Knight Diagnostics) and a 182 gene panel (Foundation One next generation panel) in selected patients. In addition, our patients all had advanced disease with multiple prior therapies. While the number and type of prior therapies could have an effect on the evolution of type and pattern of mutation, our study did not reveal a pattern, though a larger group may be needed for a robust analysis of specific associations. Shukla et al. [17] found *BRAF*, *NRAS* and *CTNNB1* mutations in their ES patients and cell lines, while we found *KRAS*, *PTPRD*,*GRB10* and *MET*. Together, these results suggest that advanced and resistant ES (and DSRCT) may be driven by diverse secondary aberrations.

The specific genetic aberrations and their correlation with response and resistance may be also illuminating. For instance, patient 1 (Table 2) achieved a PR on combined IGF1R and mTOR inhibitor therapy lasting six months. In that patient, tissue was available (both pre- and post-treatment tumors) and only the post-treatment tumor showed a *KRAS* mutation. This result suggests an adaptive mechanism of resistance to IGF1R/mTOR attributable to the RAS/RAF/MEK pathway (Figure 2) and is consistent with those shown previously by morphoproteomic analysis where resistance to IGF1R/mTOR inhibitor was associated with ERK activation [11]. An impact of the *KRAS* mutation was confirmed in this patient's tumor tissue by immunohistochemistry-based morphoproteomics which demonstrated that the Ras/Raf kinase/extracellular signal-regulated kinase (ERK) pathway was constitutively activated with chromogenic signal observed, up to 3+ in nucleus and ± in the cytoplasm, for p-ERK 1/2 (Thr 202/Tyr 204) (Figure 3). Acquired *KRAS* mutations have also been implicated as a mechanism of resistance after anti-EGFR targeted therapy in colorectal cancer. [27], [28], [29], [30]. In addition, it has been shown that the emergence of *KRAS* mutant clones can be detected months before radiographic progression [27], [29]. In colorectal cancer, it has been suggested that early initiation of a MEK inhibitor may be a rational strategy for delaying or reversing drug resistance [27], [29]. While it was not possible to provide this particular patient with a MEK inhibitor concurrently with combined IGF-1R/mTOR inhibition, due to constraints inherent in clinical trials, joint blockade of the MAPK and PI3K/Akt/mTOR cascades +/− IGF-1R inhibition may be an interesting approach for patients who acquire resistance to IGF-1R/mTOR-based therapies.

**Table 2:** Characteristics of Ewing's sarcoma and DSRCT patients with somatic mutations.

Patient* #	Age at Dx	Pathology/fusion type	Best Reponse to IGF1R inhibitor	Best Response to IGF1R+mTOR inhibitor	Mutation	Time of tumor sample
**1**	16	CD99+;EWSR1(22q12)	N/A	**PR**	KRAS G13N (c.37_38GG>AA)	Tumor resistant to IGF1R+mTOR inhibitor (Pre-treatment sample did not show KRAS mutation)
**2**	24	CD99+;EWSR1(22q12)	**CR**	**CR**	PTPRD W775stop GRB10 Q107stop	Tumor resistant to IGF1R inhibitor
**3**	13	CD99+;EWSR1(22q12)	N/A	**CR**	GRB10 V109A	Before treatment with IGF1R inhibitor
**4**	20	CD99+;EWSR1(22q12)	N/A	N/A	MET T1010I	
**19**	13	EWSR1-WT1	N/A	N/A	MET N375S	
**20**	52	EWSR1-WT1	N/A	N/A	PIK3CA M1040I, G1049S	

* Patient numbers according to Table 1.

Abbreviations: CR- Complete remission, PR- Partial remission,N/A-Not applicable.

Patient 2 (ES) (Table 1) initially received IGF1R antibody treatment and achieved a CR for nearly three years before developing resistance [11]. After subsequent enrollment in a study of combined IGF1R/mTOR inhibition, she achieved a second CR that lasted two years. Two mutations were identified in her tumor sample collected between the two lines of treatment, a *GRB10* Q107stop mutation and a *PTPRD* W775 stop mutation. The latter mutation results in a truncated non-functional protein product incapable of dephosphorylating STAT3 [25]. (Of interest, STAT3 phosphorylation is found in about 50% of Ewing's sarcomas [22].) The loss of PTPRD function would be expected to suppress STAT3 dephosphorylation and lead to accumulated phosphorylated STAT3, especially under conditions of activated p-IGF1R [26], [31] (Figure 2). One could reasonably speculate that patient 2 responded to IGF1R therapy, even in the presence of a mutated PTPRD, because p-STAT3 remains critically dependent on intact IGF-1R signaling for downstream effects.

As mentioned, patient 2 (Table 2) also had a *GRB10* mutation. The growth factor receptor-bound protein 10 (Grb10) is reported to be an mTORC1 substrate that acts downstream of IGF1R pathway [23], [24]. The mTORC1 protein regulates the phosphorylation of Grb10 at S501 and S503, which indirectly results in the inhibition of PI3K, and thus regulates the IGF1R pathway through a negative feedback [24] (Figure 2). Mutation of Q107 to a stop codon, as found in this patient, would be expected to lead to a truncated Grb10 protein and loss of the phosphorylation sites on Grb10. This, in turn, would be expected to abrogate the negative feedback regulation of Grb10 and result in enhanced IGF1R pathway activity. Among a number of possible mechanisms of acquired resistance, this mutation may therefore have contributed to the eventual development of resistance to the IGF1R inhibitor treatment.

Patient 3 (ES) (table 1) achieved a CR, which lasted for two years in response to a combination regimen using an IGF1R and an mTOR inhibitor. This patient was specifically studied for a GRB10 mutation because of the similarity in his outcome to that of patient #2. A *GRB10* V109A mutation was identified in his tumor tissue, which would not be expected to abrogate GRB10 function as the Q107 stop mutation previously noted in patient 2. However, it is possible that the V109A mutation resulted in a sufficient conformational change of the Grb10 protein to impair the negative feedback to the IGF1R pathway.

In addition to the mutations in *GRB10* and *PTPRD* that can directly affect IGF-1R signaling, an unexpected mutation in MET was identified in patients with ES and DSRCT. Patient 4 (ES, Table 2) demonstrated a *MET* T1010I mutation, which has been reported in lung cancer patients [32] but never in ES. MET belongs to the receptor tyrosine kinase (RTK) family and has been known to stimulate cancer cell growth [33]. The T1010I mutation is located in the juxtamembrane domain, which is essential for catalytic function of receptor tyrosine kinases [34]. The mutation may compromise the negative feedback function of the MET pathway, and thus lead to tumor growth. Patient 19 (DSRCT, Table 2) also demonstrated a *c-MET* N375S mutation in his tumor tissue. This is the most frequent mutation in *MET* and has primarily been reported in patients with lung cancer. However, this is the first time that the mutation has been reported in a patient with DSRCT. The N375S mutation has been previously observed as contributing to the resistance of MET to its inhibitors [35].

Patient 20 (DSRCT, Table 2) demonstrated two *PIK3CA* mutations, M1040I and G1049S, both located in the kinase domain of p110α near the activation loop [36]. The protein product expressed by *PIK3CA* is p110α, which forms a PI3K complex with p85α [37]. The PI3K/AKT/mTOR pathway regulates cell proliferation and tumor growth [38]. The M1040I and G1049S mutations are located in the kinase domain of p110α, near the activation loop [36]. H1047 mutations have been reported to result in enhanced kinase activity [39], one may hypothesize that M1040 and G1049 mutations located in the same helix with H1047 would similarly bolster the kinase activity of p110α by inducing an active conformation of the kinase activation loop. Yet, the precise role of these mutations in ES or DSRCT as well as the effectiveness of PI3K inhibitors remains to be validated.

There are several important limitations to this study. First, it is a descriptive study. Indeed, the analysis was largely retrospective with a variety of different methods used for molecular profiling. Second, the patients selected for in-depth molecular assessment were enriched for those who had responded to biologically-targeted therapy. Third, there was a lack of matched normal tissue, so it is unclear to what extent some of these aberrations might be somatic versus germ-line. Fourth, there were limitations in tissue availability and an evolution in the technologies used to analyze molecular features. As a result, while our research identified the emergence of a number of novel mutations in ES and DSRCT, their frequency and association with response requires additional study. Lastly, a series of controlled experiments are needed to test and validate the functional impact of some of these aberrations. Our findings should therefore be viewed as hypothesis generating.

In summary, we have reported diverse secondary aberrations in advanced ES and DSRCT. Some of these aberrations may be actionable and, therefore, potentially have implications for molecularly-directed targeted therapy. As the technology to assess whole genomes, transcriptomes and more is evolving rapidly, it is likely that these techniques will be increasingly applied, and may reveal even more complex molecular portfolios for patients with metastatic Ewing's sarcoma and desmoplastic small round cell tumor.
